# Improved Learning in U.S. History and Decision Competence with Decision-Focused Curriculum

**DOI:** 10.1371/journal.pone.0045775

**Published:** 2012-09-21

**Authors:** David Jacobson, Andrew Parker, Chris Spetzler, Wandi Bruine de Bruin, Keith Hollenbeck, David Heckerman, Baruch Fischhoff

**Affiliations:** 1 Administration, Springfield School District, Springfield, Oregon, United States of America; 2 RAND Corporation, Pittsburgh, Pennsylvania, United States of America; 3 Decision Education Foundation, Palo Alto, California, United States of America; 4 Department of Engineering and Public Policy, Carnegie Mellon University, Pittsburgh, Pennsylvania, United States of America; 5 College of Education, University of Oregon, Eugene, Oregon, United States of America; 6 Microsoft Research, Los Angeles, California, United States of America; 7 Department of Social and Decision Sciences, Department of Engineering and Public Policy, Carnegie Mellon University, Pittsburgh, Pennsylvania, United States of America; University of Sheffield, United Kingdom

## Abstract

Decision making is rarely taught in high school, even though improved decision skills could benefit young people facing life-shaping decisions. While decision competence has been shown to correlate with better life outcomes, few interventions designed to improve decision skills have been evaluated with rigorous quantitative measures. A randomized study showed that integrating decision making into U.S. history instruction improved students’ history knowledge and decision-making competence, compared to traditional history instruction. Thus, integrating decision training enhanced academic performance and improved an important, general life skill associated with improved life outcomes.

## Introduction

Better decision making is crucial to improving lives. Indeed, higher scores on standardized tests of decision-making competence correlate with better life outcomes [1]–[3]. Although long taught at the university level, decision making is rarely part of high school curricula [4], where its inclusion could benefit young people facing decisions that influence the course of their lives (e.g., about education, careers, and health behaviors).

One way to accommodate decision making in the high-school curriculum is to integrate it in existing courses. For example, history students could examine historical events in terms of the choices facing historical figures. We report on an experiment that integrated decision training into high school U.S. History courses in an effort to improve both history knowledge and decision-making competence. Analogous approaches could be pursued with science, math, or English courses.

The study involved five teachers at a large comprehensive high school (1490 students) in the Pacific Northwest. Two teachers integrated approximately 15 hours of decision skills curriculum in school-district-approved U.S. history curriculum. In their courses, students analyzed historic decisions in terms of the goals, alternatives, uncertainties, and critical tradeoffs historic figures faced. Two experimental group teachers developed decision skills materials using the Decision Quality framework developed by the Decision Education Foundation [5]. Three control group teachers delivered the standard history curriculum.

All sophomores were randomly assigned to experimental group classes receiving the decision-focused history curriculum, or to control classes receiving the standard history curriculum, stratifying by (a) gender, (b) socio-economic status, (c) reading scores and (d) grade point average. Overall, there were four experimental and twelve control group classes with 100 experimental and 178 control group students.

Students were tested at the beginning and the end of the two-term course. History knowledge was assessed with 42 retired questions from the National Assessment of Educational Process (NAEP) U.S. history test (see Text S1). Decision skills were assessed with the Decision-Making Competence (DMC) test [1], modified to exclude questions about sensitive risk behaviors (see Text S2). Pre-and posttests were identical in content, format, and administration, at both times and for both groups. Students were notified that the district was evaluating the history curriculum, but not about the experiment.

## Results

A t-test showed no statistical differences between the Experimental and Control groups on the NAEP pretest (*p* = 0.23). The mean scores for the NAEP pretest (with standard deviations in parentheses) for the Experimental Group and the Control groups were 18.19 (5.35) and 17.41 (5.38), respectively. Similarly, no statistical differences existed between the Experimental and Control groups on the DMC pretest (*p* = 0.11). The scores for the DMC pretest for the Experimental group and the Control group were 0.01 (0.42) and −0.09 (0.51), respectively.

For the NAEP, observed posttest means (standard deviations) for the Experimental group and the Control group were 25.97 (6.01), and 23.78 (6.60), respectively. Divided by the 42 NAEP questions, the difference of the means (2.19) represents a 5.2% improvement, equivalent to half a standardized grade [6]. For the DMC, posttest means (with standard deviations in parentheses) for the Experimental group and the Control group were 0.14 (0.42) and −0.01 (0.49), respectively.

**Figure 1:**
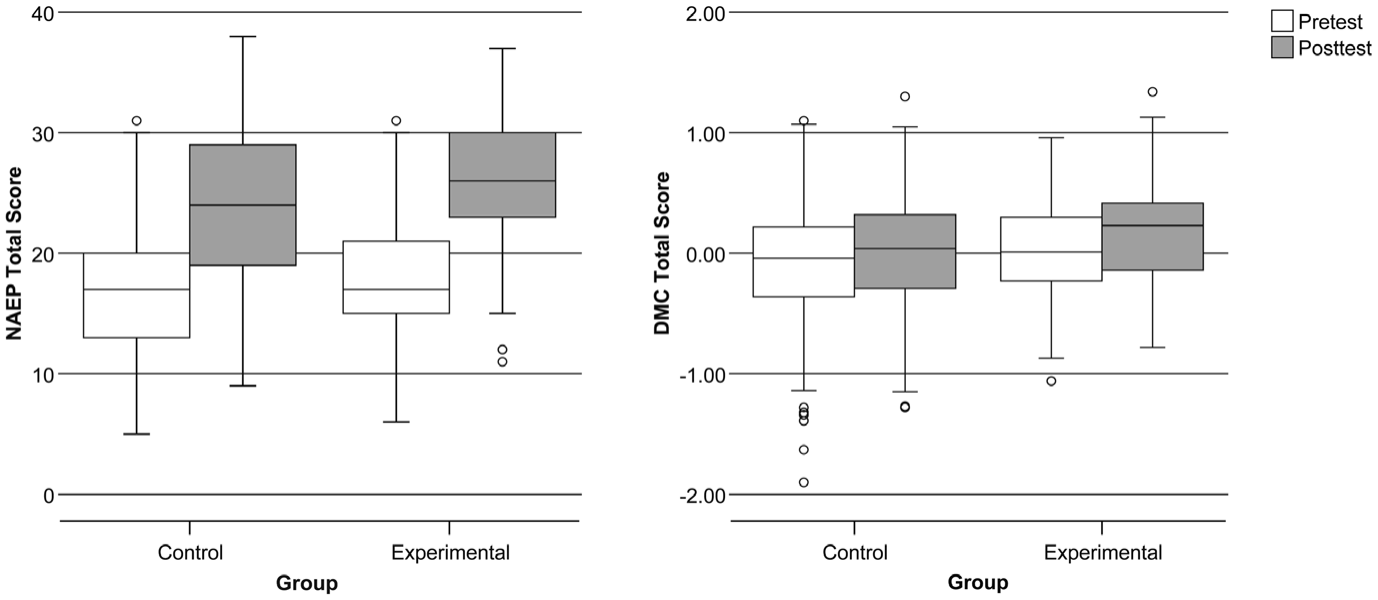
Summary statistics for history knowledge (NAEP) and decision-making competence (DMC) scores at pre- and post-test.

Figure 1 presents pretest and posttest scores for history knowledge (NAEP) and decision-making skills (DMC). History knowledge showed significantly greater improvement in the Experimental group, which received the decision-focused curriculum, than in the Control group, which received the standard curriculum (p = 0.008). Decision-making skills also improved significantly more in the Experimental group than in the Control group, (p = 0.015). The Experimental group showed improvements in NAEP even when those scores were adjusted for improvements in DMC (p = 0.010), and vice-versa (p = 0.019).

## Discussion

Incorporating decision-making training in an otherwise standard history course did not distract, but rather improved academic performance (on the NAEP history test) as well as decision skills (on the DMC test). The five percent greater improvement in history knowledge represents one-half grade point, achieved with little additional investment of time or resources.

One possible explanation for improved performance on the NAEP and DMC is that teachers in the Experimental group were better teachers. Teachers with similar experience were assigned to lead Experimental and Control classes. Further, those teaching the Experimental classes had not taught U.S. History the prior year, whereas Control group teachers had. Finally, assuming that changes in DMC and NAEP serve as indirect measures of teacher capacity, we would anticipate an interaction between the NAEP and DMC measures. However, the Experimental group showed improvements in NAEP even when those scores were adjusted for improvements in DMC, and vice-versa, suggesting that teacher quality was not responsible for the improvements. These results also indicate that improvements in decision-making skills did not influence the abilities of the students to learn history.

Another explanation is that the decision-making perspective increased students’ engagement with the history material, as reported anecdotally by the teachers and their students. Given the decision-making curriculum’s focus on problem solving and group work, this explanation is supported by recent research on “deliberate practice,” a student-centered approach that generates relevant discussion of key questions [7].

Given that decision competence was improved along with academic learning, the study provides evidence that decision skills can become a meaningful part of high school education. These preliminary results call for a larger trial, incorporating more students, teachers, and schools. The benefit of teaching decision making in different subject areas and of greater cumulative exposure to the material could be tested. Long-term follow-up could examine the persistence of gains over time and their transfer to other domains. Lasting improvements in decision-making competence may improve a student’s ability to make better decisions that would lead to better life outcomes for individuals, with downstream benefits to society as a whole.

## Methods

US History coursework covered the 1880s–1990s in two 12-week trimesters with 70 minute classes. All instructors used materials and strategies that me Oregon State Department of Education content standards.

In addition to the district-approved US History curriculum, Experimental-group participants received an integrated curriculum that included instruction in the Decision Quality (DQ) model. Exposure to the DQ material occurred in an introductory unit and was reinforced through classroom simulations, lectures, and writing assignments.

Two of five teachers were selected for the Experimental group. They were selected because, although they had taught US History previously, neither had taught US History the prior year and were willing to adjust their instructional approach. Teachers in the Experimental and Control groups had comparable experience and training. All teachers met No Child Left Behind standards for a highly qualified teacher in U.S. History. All Control-group teachers taught U.S. history courses the previous school year and had no experience with decision education.

Teachers in the experimental arm received one week of training in decision making. One of us (C.S.) worked with them to integrate existing history/decision-making course material developed by Decision Education Foundation. The teachers adapted this material and developed new material to fit their classrooms. For example, while studying Industrialization, students engaged in a classroom simulation where they took the role of steel workers deciding whether to strike for higher wages.

Students were aware the course curriculum was being studied but were not aware of the decision component of the study. All tenth graders at the school (402) participated in the study in four experimental and twelve control group classes of 25–30 students. 278 students completed both the NAEP U.S. history content and the decision competence (DMC) pretest and posttest, resulting in 178 control and 100 subjects, respectively.

Stratified random sampling placed students into Experimental and Control groups by (a) gender, (b) socio-economic status (participation in free and reduced meals), (c) eighth-grade statewide assessment reading scores (above and below mean), and (c) ninth-grade grade point average (above and below mean GPA). Study participants were 86.6% Caucasian, 7.3% Hispanic, 2.5% Native American, 1.8% African American, and 1.8% Asian/Pacific Islander.

Student content knowledge was measured with 42 multiple choice questions from the database of NAEP questions for US History. Questions addressed the historical period ranging from 1880–1980 that was delivered to all students. Pretest and posttest were identical and students were given as much time as needed to complete it. Students in both groups completed the test within one class period.

The entire DMC [1] was used to measure decision-making skills, with minor modification to ensure appropriateness for this population. The decision-making tasks include: (a) resistance to framing, (b) recognizing social norms, (c) under/overconfidence, (d) decision rules, (e) risk perception, and (f) resistance to sunk costs. The independently developed DMC aligns broadly with the DQ framework. However, the US History curriculum was focused on the lessons of DQ, and not on teaching decision-competence tasks.

A likelihood ratio test (LRT) was used to determine whether changes in NAEP scores were associated with the group variable. The null model was a regression of NAEP posttest scores on NAEP pretest scores. The alternative model consisted of two regressions of posttest scores on pretest scores, one for each group. Separate slopes and intercepts were allowed. The LRT revealed a significant difference between groups, favoring the Experimental group (*p* = 0.008). Repeating this analysis with improvement in DMC score as an additional covariate yielded a p-value of 0.010. Analogous analyses were done for DMC scores. The group variable was significantly associated with increase in DMC score whether the covariate improvement in NAEP score was excluded (p = 0.015) or included (p = 0.019).

### Ethics Statement

No medical or health related experimentation was performed. The only difference between the experimental and control group was the educational instruction received by the students. Before the study was performed, it was reviewed and approved by both the IRB of the University of Oregon and Springfield Public Schools Research Review Committee. Neither committee required informed consent of the students.

## Supporting Information

Text S1 National Assessment of Educational Process (NAEP) U.S. history test.
(PDF)

Text S2 Decision-Making Competence (DMC) test.
(PDF)
