# Supplementary material for: Improved Learning in U.S. History and Decision Competence with Decision-Focused Curriculum
Source: PLoS One. 2012 Sep 21;7(9):e45775. doi: 10.1371/journal.pone.0045775 (PMC3448680; doi:10.1371/journal.pone.0045775)
Supplement: Text S1 — National Assessment of Educational Process (NAEP) U.S. history test. (PDF) [file pone.0045775.s001.pdf]

Text S1: National Assessment of Educational Process (NAEP) U.S. history test

US History Test

Name:
Teacher: Period:

- *Brown* v. *Board of Education of Topeka*
- Montgomery Bus Boycott
- March on Washington
- "I Have a Dream" Speech
- Voting Rights Act

01 The events listed would be most important in a discussion of which movement?

A) Civil rights
B) Anti-Vietnam War
C) Immigration reform
D) Women's liberation

02 The Great Society programs of the 1960's were primarily based on the idea that

A) American society was hopelessly flawed
B) the federal government should play an active role in promoting social welfare
C) the poor needed to work harder in order to succeed
D) the federal government lacked the authority to help the poor

03 An important factor leading the United States to enter the First World War was

A) the existence of treaties between the United States, Great Britain, and Austria-Hungary
B) the United States policy of opposing communism
C) German attacks on United States shipping
D) Russian attacks on United States settlements in the Aleutian Islands

04 What does the cartoon above suggest about the attitudes of some Americans toward President Woodrow Wilson's plan for the United States to join the League of Nations?

A) Some Americans believed that his plan would violate the United States Constitution.
B) Some Americans believed that the United States was too weak to participate in the League of Nations.
C) Wilson's plan would lead the United States to lose its world leadership role.
D) Wilson's plan was unfair to the countries that lost the First World War.

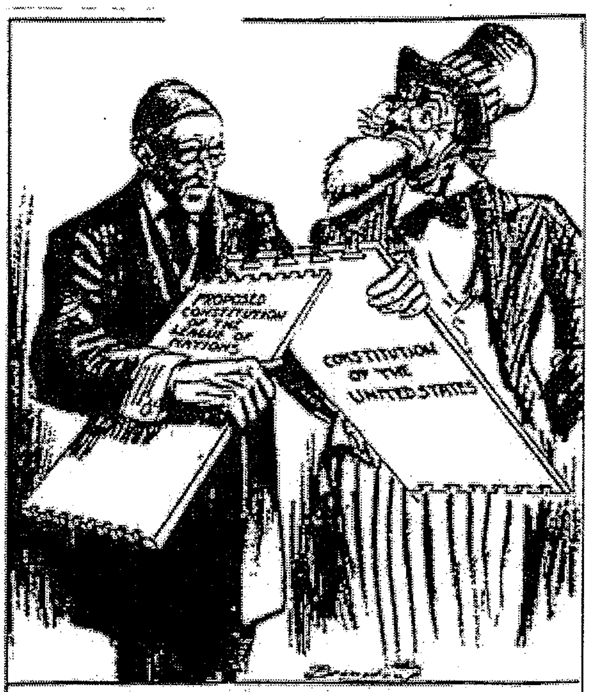

THEY WON'T DOVETAIL
—Bronstrup in San Francisco Chron

Library of Congress

05 A consequence of Prohibition was

A) the failure of the Republicans in the 1928 presidential election
B) the growth of organized crime
C) the turning of public attention to pressing international issues
D) widespread popular support for further moral reform

06 According to Document D, the prosperity of the 1920's was mainly based on

A) a strong banking sector
B) increased agricultural profits
C) an expanded volume of exports
D) the automobile and housing industries

Document D: *A modern historian's explanation of the causes of the Great Depression*

For one thing, the American economy had weakened significantly during the 1920's. The economic difficulties felt by farmers increasingly were shared by such distressed industries as coal, railroads, and textiles. As a result, by 1929, unsold inventories were stacking up, investments were shrinking, workers were being laid off, and consumers were buying fewer products.

Second, the American business system was unbalanced, with a few industries carrying the weight of the economy. Automobiles and housing construction, in particular, fueled the economic boom of the 1920's. Newer industries, such as chemicals, were still not fully developed. Thus when the market for houses and cars began to weaken, as it did in 1928, other industries were not able to take up the slack.

Third, poor distribution of purchasing power weakened the economy. Although the wages of most Americans increased in the 1920's, they grew at a much slower rate than the economy as a whole.

Fourth, an enormous amount of debt threatened the country's economic health. Farmers carried large mortgages on their land and were unable to keep up payments as their crop prices fell. Small banks, particularly the ones that had loaned money to farmers, were having serious problems as borrowers defaulted on their loans.

07 Why did the liquor industry oppose women getting the right to vote?

A) Liquor manufacturers tended to be very conservative and opposed any change in voting laws.
B) Liquor manufacturers worried that women voters would want to ban alcohol.
C) Women tended to work for liquor manufacturers, who did not want them taking time off to get involved in politics.
D) Liquor manufacturers owned "men-only" bars and worried that women would vote to make such bars illegal.

08 In which book did Upton Sinclair describe the terrible working and food-production conditions in the meat-packing industry?

A) *The Grapes of Wrath*
B) *The Pit*
C) *The Octopus*
D) *The Jungle*

09 Which war did the United States enter to prevent the spread of communism?

A) The Mexican-American War
B) The First World War
C) The Second World War
D) The Vietnam War

The following question refers to the photograph below.

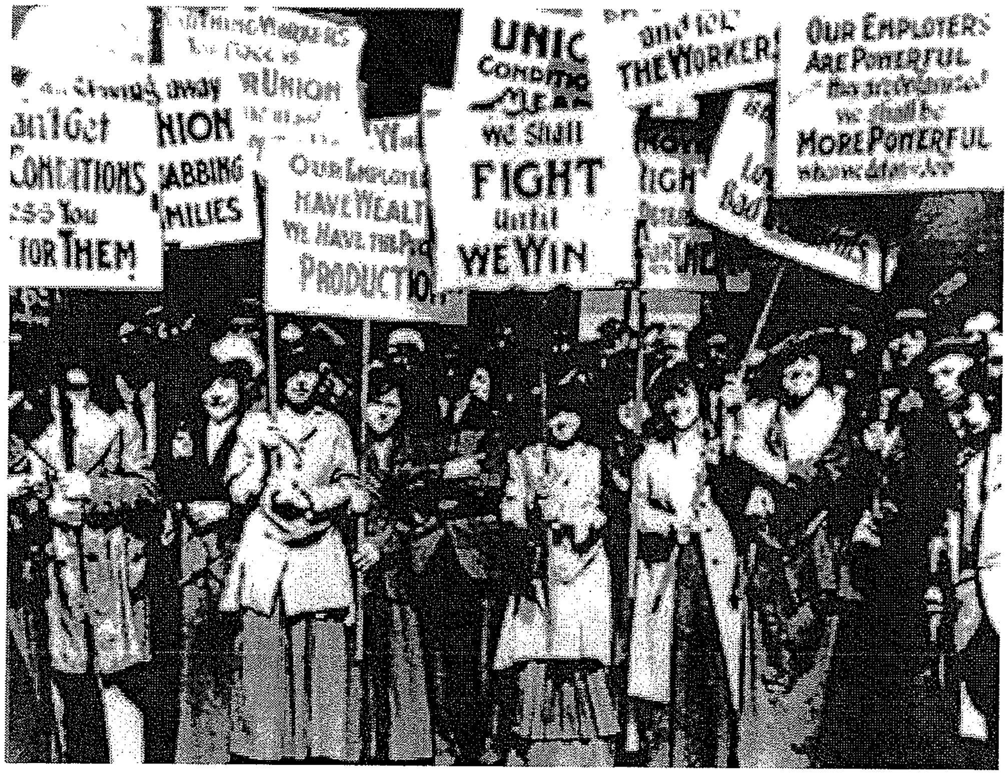

10 The photograph was probably taken in a

A) southern city in the 1880's
B) western city in the 1900's
C) northeastern city in the 1910's
D) political convention in the 1980's

The Only Way to Handle It *(Library of Congress.)*

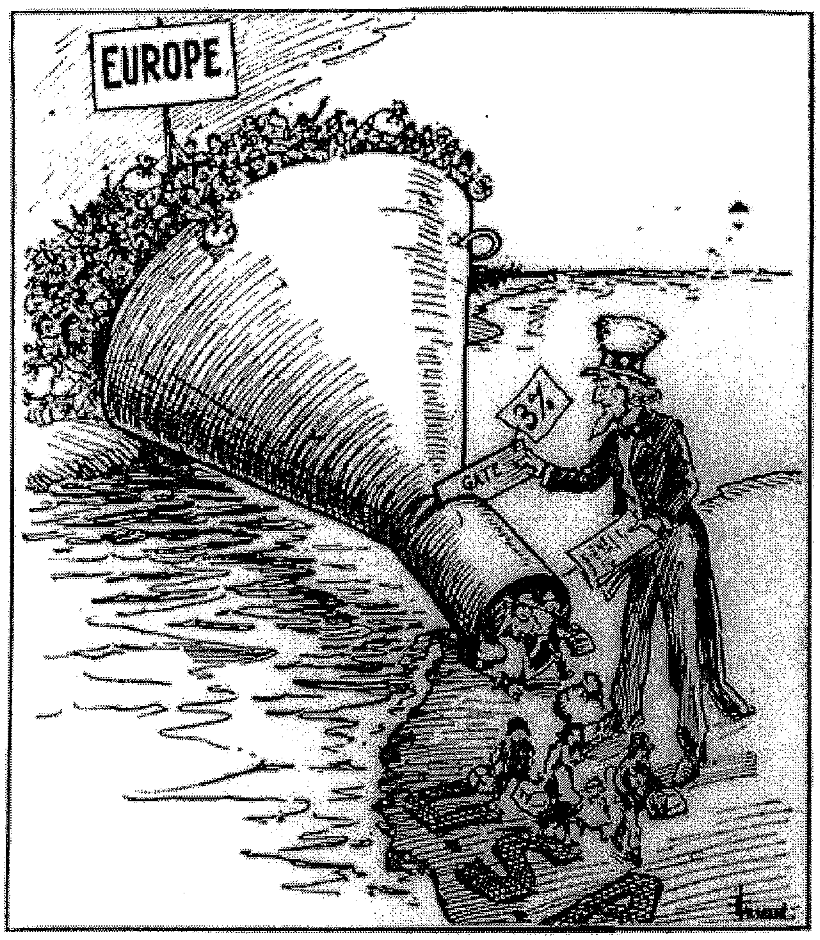

11. What does the political cartoon above show?

A) effects of a new law limiting immigration
B) The need for skilled immigrants to fill jobs created by the boom after the First World War
C) The desire of reformers to provide social services for European immigrants
D) The eagerness of the government to settle immigrants inland rather than in the eastern industrial cities

12 The Monroe Doctrine, the Good Neighbor Policy, and the Alliance for Progress were all United States foreign policy positions relating directly to

A) Latin America
B) Africa
C) the Middle East
D) China

13 When the United States entered the Second World War, one of its allies was

A) Germany
B) Japan
C) the Soviet Union
D) Italy

14 Documents G and H show that Roosevelt's highest priority was reducing

A) unemployment
B) trade imbalances
C) food prices
D) stock prices

Document G: *Excerpt from Franklin D. Roosevelt's First Inaugural Address (1933)*

This great Nation will endure as it has endured, will revive and will prosper. So, first of all, let me assert my firm belief that the only thing we have to fear is fear itself--nameless, unreasoning, unjustified terror which paralyzes needed efforts to convert retreat into advance. A host of unemployed citizens face the grim problem of existence, and an equally great number toil with little return. Our greatest primary task is to put people to work. This is not an unsolvable problem if we face it wisely and courageously. It can be accomplished in part by direct recruiting by the Government itself . . . accomplishing greatly needed projects to stimulate and reorganize the use of our natural resources.

Document H: *Key New Deal Legislation*

Year	***Act or Provisions***	***Provisions***
1933	Agricultural Adjustment Administration (AAA)	Granted farmers direct payments for reducing crop production; funds for payment provided by a processing tax, later declared unconstitutional.
1933	Tennessee Valley Authority (TVA)	Constructed dams and power projects and developed the economy of a nine-state area in the Tennessee River Valley
1933	National Industrial Recovery Act (NIRA)	Sought to revive business through a series of fair competition codes; created National Recovery Administration (NRA) to write, coordinate, and implement these codes; NIRA's Section 7a guaranteed labor's right to organize (act later declared unconstitutional).
1933	Public Works Administration (PWA)	Sought to increase employment and business activity through construction of roads, buildings, and other projects.
1933	Federal Emergency Relief Act (FERA)	Provided federal funds for state and local relief efforts.
1933	Civil Works Administration (CWA)	Provided federal jobs for the unemployed.
1933	Civilian Conservation Corps (CCC)	Employed young men in reforestation, road construction, and flood control projects.
1934	Federal Housing Administration (FHA)	Insured loans provided by banks for the building and repair of houses.
1935	Social Security Act	Created a system of social insurance that included unemployment compensation and old age survivors' insurance; paid for by a joint tax on employers and employees.
1935	Works Progress Administration	Employed more than eight million people to repair roads, build bridges, and work on other projects; also hired artists and writers.
1935	National Youth Administration (NYA)	Provided job training for unemployed youths and part-time jobs for students in need.
1938	Fair Labor Standards Act	Established a minimum wage of 40 cents an hour and a maximum workweek of 40 hours for businesses engaged in interstate commerce.

15 Which sentence states what most New Deal supporters thought the federal government should do?

A) The federal government should protect business in order to increase productivity.
B) The federal government should protect the rights and interests of minority groups.
C) The federal government should regulate and directly stimulate the economy by spending and hiring.
D) The federal government should play as small a role as possible in the economy.

16 In Document K, Huey Long is criticizing President Roosevelt for his

A) foreign policy
B) agricultural policy
C) Civilian Conservation Corps
D) attacks on the rich

Document K: *Excerpt from a speech given by Huey Long, March 7, 1935*

So it has been while millions have starved and gone naked and while babies have cried and died wanting milk; so it has been while people begged for meat and bread to eat. Mr. Roosevelt's administration has sailed merrily along, plowing under and destroying the things to eat and wear, with tear-dimmed eyes and hungry souls made to chant for this New Deal so that even their starvation dole is not taken away from them, and meanwhile the food and clothes craved by humanity for their bodies and souls go to destruction and ruin.

17 "Imagine a six-foot-deep ditch weaving from Washington, D.C., to Detroit, Michigan. Men's heads could not be seen over the top edges. Dugouts would be tunneled for sleeping and eating quarters."

The passage above describes battle conditions first experienced by United States soldiers fighting in the

A) First World War
B) Second World War
C) Korean War
D) Vietnam War

18 In Montgomery, Alabama, African Americans were required by law to sit in the back of city buses. From 1955 to 1956, African Americans organized a bus boycott, refusing to ride on the city buses. Why was the Montgomery bus boycott important?

A) It helped African American workers to get higher pay.
B) It helped begin the modern civil rights movement.
C) It forced southern bus lines to close.
D) It led people to be more conscious of conserving energy.

The following question refers to two photographs. The first photograph shows a sign in front of a laundry that states "We wash for white people only." The second photograph shows a sign stating "For Whites Only" next to a picnic area at a beach.

19 The photographs show

A) prejudice
B) poverty
C) political protest
D) environmental conservation

20 President Franklin D. Roosevelt's goal in supporting the Lend-Lease Act of 1941 was to

A) encourage Japanese Americans to relocate voluntarily
B) use foreign investment as a way of stimulating the American economy
C) maintain an isolationist stance by providing only limited aid to both sides in the European conflict
D) assist Britain's war effort without violating United States neutrality laws

21 What goal was most important in shaping United States foreign policy between 1945 and 1990?

A) Preventing the spread of communism to new areas and weakening it where it already existed
B) Encouraging trained scientists and other skilled workers who lived in foreign countries to immigrate to the United States
C) Strengthening the United States industrial and agricultural sectors to help them compete against the British and the French
D) Providing foreign aid to all poor countries to help them develop economically and technologically

The following question refers to the headline and newspaper cartoon below.

SOVIETS LAUNCH FIRST MAN-MADE SATELLITE INTO ORBIT

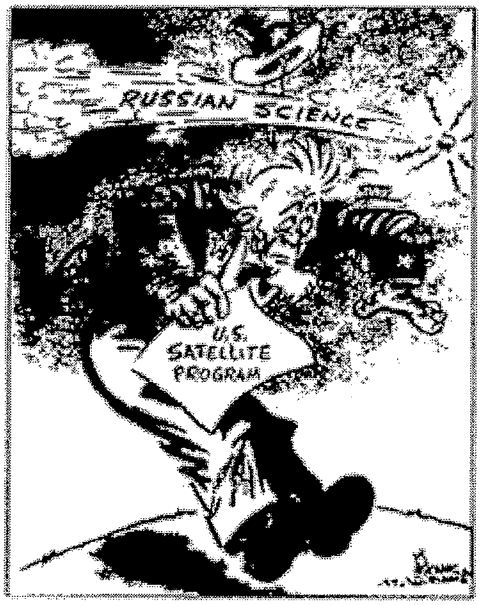

Frank Williams in the Detroit Free Press.

22 What did the United States government do in response to the event referred to in the cartoon and headline?

A) The government decided to seek peace immediately and to end the Cold War.
B) The government banned civilian contact between United States and Soviet citizens.
C) The government decided to spend more on both scientific education and the military.
D) The government requested that the United Nations prohibit Soviet space exploration.

IT CERTAINLY WAS LOADED!

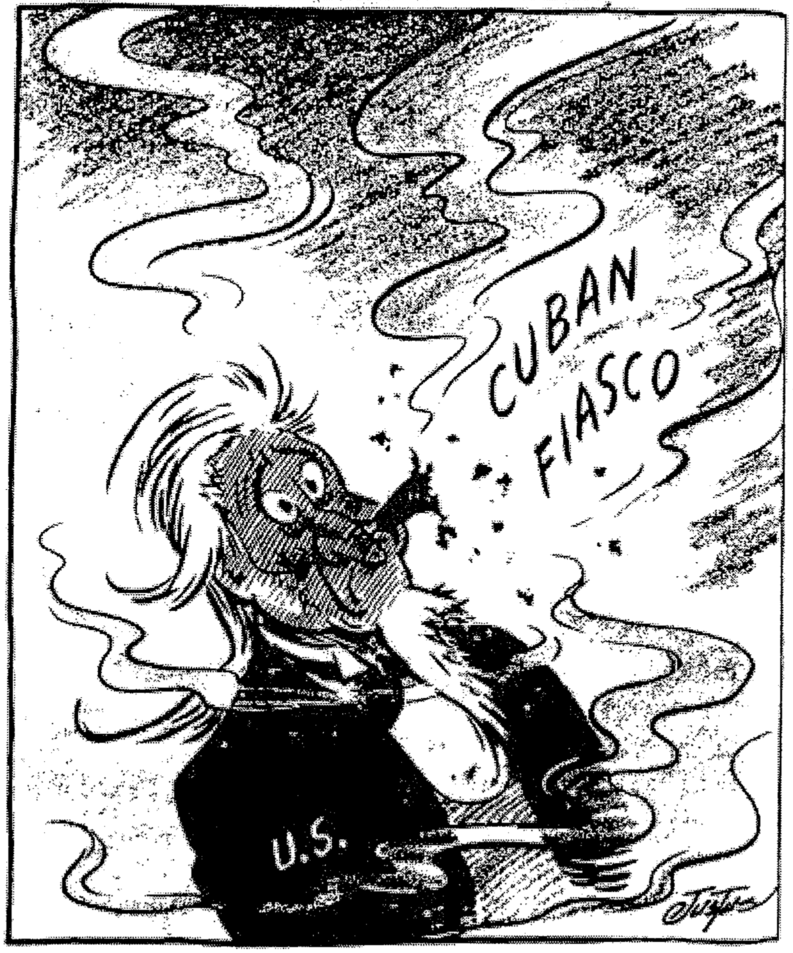

Roy Justus, Minneapolis Star Tribune.
Reprinted with permission.

23 The cartoon above depicts United States frustration with

A) the Good Neighbor policy
B) Dollar Diplomacy
C) the Spanish-American War
D) the Bay of Pigs invasion

24 The Gulf of Tonkin Resolution (1964) was significant because it

A) ended the war in Korea
B) gave President Johnson the authority to expand the scope of the Vietnam War
C) was an attempt to take foreign policy power away from the President
D) allowed China to become a member of the United Nations

25 The phrase "Harlem Renaissance" refers to

A) African American political gains during the Reconstruction period
B) African American achievements in art, literature, and music in the 1920's
C) religious revival in the African American community that swept the nation in the 1950's
D) a series of urban renewal projects that were part of the Great Society program of the 1960's

26 When the United States entered the Second World War, one of its allies was

A) Germany
B) Japan
C) the Soviet Union
D) Italy

27 Many writers of the "lost generation," such as F. Scott Fitzgerald, wrote books in which they

A) criticized what they regarded as the shallow materialism of the United States during the 1920's
B) sought to capture the freedom experienced by people living on the western frontier
C) praised the quick pace and unique opportunities found in large cities in the United States
D) expressed deep yearning for what they regarded as the simple lives led by rural Americans

The following question refers to the following cartoon.

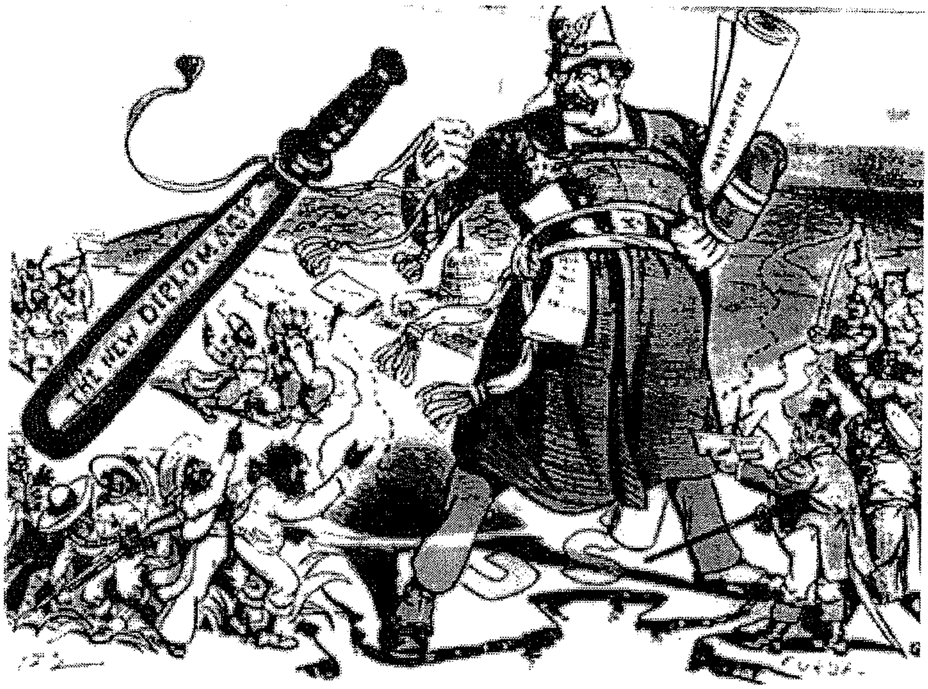

Cartoon by Louis Dalrymple.

28 When the cartoon was created, the artist was probably thinking of which foreign policy slogan?

A) The arsenal of democracy
B) Peace with honor
C) We have nothing to fear but fear itself
D) Speak softly and carry a big stick

29 Which sentence best states the central belief of late nineteenth-century Social Darwinists?

A) Members of any species survive in large part because of cooperation among members of that species, and humans should follow this natural model.
B) All men and women are created with equal abilities and should, therefore, have equal rights.
C) Modern forms of social organization, work, and family life have alienated workers from their labor.
D) Some people or nations are more fit to survive than others and will naturally and rightly dominate.

30 The urban population explosion in the late 1800's was primarily a result of

A) immigration
B) rapid increases in family size
C) dramatic reductions in the urban death rate
D) African American migration to the North and West

31 In the 1970's the United States economy was directly affected by

A) a sharp increase in the price of oil
B) an increase in the cost of solar energy
C) an overall decline in international trade
D) a rapid decline in prices of consumer goods

32 President Jimmy Carter played a major role in negotiating the Camp David accords, which promoted peace between

A) the Soviet Union and China
B) the Palestinians and the Jordanians
C) Egypt and Israel
D) North Korea and the United States

33 The opening of diplomatic relations between the United States and China's communist government occurred during the presidential administration of

A) Harry S Truman
B) John F. Kennedy
C) Lyndon B. Johnson
D) Richard M. Nixon

34 To achieve his goals, Martin Luther King, Jr., told people to

A) peacefully disobey laws that were not fair
B) ignore politics and participate in religion
C) move away from countries where they were not treated fairly
D) protest by not voting

35 The North Atlantic Treaty Organization (NATO) and the Warsaw Pact are best described as two

A) organizations founded by the European Economic Community to promote trade between Europe and the United States
B) treaties negotiated between the Allies and the Central Powers at Versailles after the First World War
C) bodies established by the United Nations to promote peace within multiethnic European countries such as Czechoslovakia and Yugoslavia
D) military organizations made up, respectively, of the United States and its allies and the Soviet Union and its allies during the Cold War

The following question refers to the quotation below.

> It must be the policy of the United States to support free peoples who are resisting . . . armed minorities or . . . outside pressures.
>
> \- Harry S Truman, 1947

36 The policy described was part of a larger policy of the Truman administration that was referred to as

A) nativism
B) massive retaliation
C) isolationism
D) containment

37 In October 1962 the United States and the Soviet Union came close to war over the issue of Soviet

A) control of East Berlin
B) missiles in Cuba
C) support of the Ho Chi Minh regime in Vietnam
D) military support of the Marxist regime in Afghanistan

38 Based on the graphs and your knowledge of history, you can conclude that the first successful steps toward partial recovery from the Great Depression occurred

A) at the end of the First World War
B) with the passage of the Hawley-Smoot Tariff
C) with United States entry into the Second World War
D) with the election of Franklin Delano Roosevelt as President

<u>Document E</u>: *Charts and graphs showing economic information about the Great Depression*

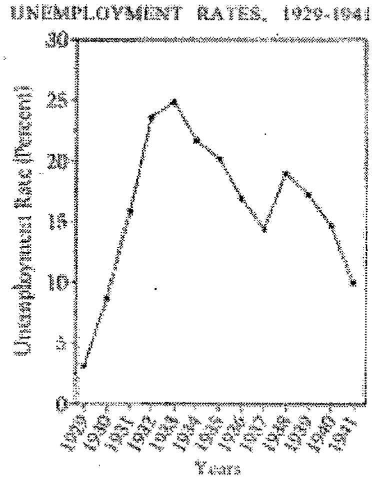

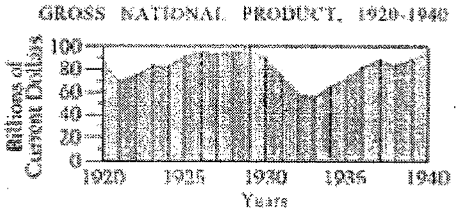

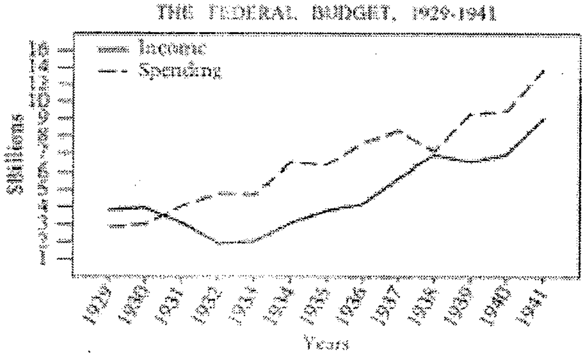

39 In 1935 and 1936 the Supreme Court declared that important parts of the New Deal were unconstitutional. President Roosevelt responded by threatening to

A) impeach several Supreme Court justices
B) eliminate the Supreme Court
C) appoint additional Supreme Court justices who shared his views
D) override the Supreme Court's decisions by gaining three-fourths majorities in both houses of Congress

The following question is based on the graph below.

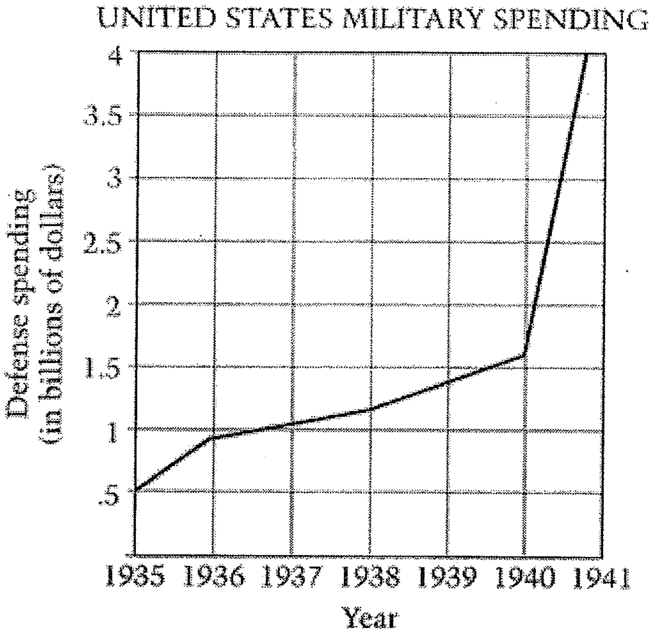

40 Which event caused the largest change in military spending shown in the graph?

A) The First World War
B) The Second World War
C) The Vietnam War
D) The Korean War

41 The Lend-Lease Act, the Yalta Conference, and the dropping of the atomic bomb on Hiroshima are all associated with the

A) First World War
B) Second World War
C) Korean War
D) Vietnam War

The following question refers to the statement below.

> We now know what we should have known then—not only was the evacuation wrong but Japanese Americans were and are loyal Americans.
>
> - Gerald R. Ford, 1976

42 The "evacuation" that Ford refers to directly affected

A) Japanese Americans and German Americans
B) Japanese citizens living on the East Coast
C) United States citizens of Japanese descent
D) Japanese soldiers serving in the United States Army
