# Supplementary material for: Improved Learning in U.S. History and Decision Competence with Decision-Focused Curriculum
Source: PLoS One. 2012 Sep 21;7(9):e45775. doi: 10.1371/journal.pone.0045775 (PMC3448680; doi:10.1371/journal.pone.0045775)
Supplement: Text S2 — Decision-Making Competence (DMC) test. (PDF) [file pone.0045775.s002.pdf]

Text S2: Decision-Making Competence (DMC) test

Name:	***Teacher:***
RC1	***Period:***

Instructions:
Each of the following problems presents a choice between two options. Each problem is presented with a scale ranging from 1 (representing one option) through 6 (representing the other option). For each item, please circle the number on the scale that best reflects your relative preference between the two options.

Problem 1

Imagine that recent evidence has shown that a pesticide is threatening the lives of 1,200 endangered animals. Two response options have been suggested:

If Option A is used, 600 animals will be saved for sure.

If Option B is used, there is a 75% chance that 800 animals will be saved, and a 25% chance that no animals will be saved.

Which option do you recommend to use?

1	2	3	4	5	6
Definitely would choose A					*Definitely would choose B*

Problem 2

Because of changes in tax laws, you may get back as much as $1200 in income tax. Your accountant has been exploring alternative ways to take advantage of this situation. He has developed two plans:

If Plan A is adopted, you will get back $400 of the possible $1200.

If Plan B is adopted, you have a 33% chance of getting back all $1200, and a 67% chance of getting back no money.

Which plan would you use?

1	2	3	4	5	6
Definitely would choose A					*Definitely would choose B*

Problem 3

Imagine that in one particular state it is projected that 1000 students will drop out of school during the next year. Two programs have been proposed to address this problem, but only one can be implemented. Based on other states' experiences with the programs, estimates of the outcomes that can be expected from each program can be made. Assume for purposes of this decision that these estimates of the outcomes are accurate and are as follows:

If Program A is adopted, 400 of the 1000 students will stay in school.

If Program B is adopted, there is a 40% chance that all 1000 students will stay in school and 60% chance that none of the 1000 students will stay in school.

Which program would you favor for implementation?

1	2	3	4	5	6
Definitely would choose A					*Definitely would choose B*

Problem 4

Imagine that the U.S. is preparing for the outbreak of an unusual disease, which is expected to kill 600 people. Two alternative programs to combat the disease have been proposed. Assume that the exact scientific estimates of the consequences of the programs are as follows:

If Program A is adopted, 200 people will be saved.

If Program B is adopted, there is a 33% chance that 600 people will be saved, and a 67% chance that no people will be saved.

Which program do you recommend to use?

1	2	3	4	5	6
Definitely would choose A					*Definitely would choose B*

Problem 5

Imagine that your doctor tells you that you have a cancer that must be treated. Your choices are as follows:

Surgery: Of 100 people having surgery, 90 live through the operation, and 34 are alive at the end of five years.

Radiation therapy: Of 100 people having radiation therapy, all live through the treatment, and 22 are alive at the end of five years.

Which treatment would you choose?

1	2	3	4	5	6
Definitely would choose surgery					*Definitely would choose radiation*

Problem 6

Imagine that your client has $6,000 invested in the stock market. A downturn in the economy is occurring. You have two investment strategies that you can recommend under the existing circumstances to preserve your client's capital.

If strategy A is followed, $2,000 of your client's investment will be saved.

If strategy B is followed, there is a 33% chance that the entire $6,000 will be saved, and a 67% chance that none of the principal will be saved.

Which of these two strategies would you favor?

1	2	3	4	5	6
Definitely would choose A					*Definitely would choose B*

Name: RC1

Teacher:
Period:

Problem 7

Imagine a hospital is treating 32 injured soldiers, who are all expected to lose one leg. There are two doctors that can help the soldiers, but only one can be hired:

If Doctor A is hired, 20 soldiers will keep both legs.

If Doctor B is hired, there is a 63% chance that all soldiers keep both legs and a 37% chance that nobody will save both legs.

Which doctor do you recommend?

1	2	3	4	5	6
Definitely would choose A					*Definitely would choose B*

Instructions:
Each of the following problems ask you to rate your judgment of a product or a situation. Each problem is presented with a scale ranging from 1 (representing the worst rating) through 6 (representing the best rating). For each problem, please circle the number on the scale that best reflects your judgment.

Problem 1

Imagine that a type of pill has a 95% success rate. That is, if you have contact with someone who has the swine flu virus, there is a 95% chance that this type of pill will prevent you from being catching the swine flu virus.

Should the government allow this type of pill to be advertised as "an effective method for lowering the risk of swine flu?"

1	2	3	4	5	6
Definitely no					*Definitely yes*

Problem 2

Imagine the following situation. You are entertaining a special friend by inviting them for dinner. You are making your favorite lasagna dish with ground beef. Your roommate goes to the grocery store and purchases a package of ground beef for you. The label says 80% lean ground beef.

What's your evaluation of the quality of this ground beef?

1	2	3	4	5	6
Very low					*Very high*

Problem 3

In a recent confidential survey completed by graduating seniors, 35% of those completing the survey stated that they had never cheated during their high school career.

Considering the results of the survey, how would you rate the incidence of cheating at your high school?

1	2	3	4	5	6
Very low					*Very high*

Problem 4

As manager, one of your project teams has come to you requesting an additional $100,000 in funds for a project you instituted several months ago. The project is already behind schedule and over budget, but the team still believes it can be successfully completed. You currently have $500,000 remaining in your budget unallocated, but which must carry you for the rest of the fiscal year. Lowering the balance by an additional $100,000 might jeopardize flexibility to respond to other opportunities.

Evaluating the situation, you believe there is a fair chance the project will not succeed, in which case the additional funding would be lost; if successful, however, the money would be well spent. You also noticed that of the projects undertaken by this team, 30 of the last 50 have been successful.

What is the likelihood you would fund the request?

1	2	3	4	5	6
Very unlikely					*Very likely*

Problem 5

Suppose a student got 90% correct in the mid-term exam and 70% correct in the final-term exam, what would be your evaluations of this student's performance?

1	2	3	4	5	6
Very poor					*Very good*

Problem 6

Imagine that a woman parked illegally. After talking to her, you believe that there is a 20% chance that she did not know she parked illegally.

With this in mind, how much of a fine do you believe this woman deserves?

1	2	3	4	5	6
Minimum fine					*Maximum fine*

Problem 7

Imagine that a new technique has been developed to treat a particular kind of cancer. This technique has a 50% chance of success, and is available at the local hospital.

A member of your immediate family is a patient at the local hospital with this kind of cancer. Would you encourage him or her to undergo treatment using this technique?

1	2	3	4	5	6
Definitely no					*Definitely yes*

<u>Instructions:</u>
The following problems ask whether it is sometimes OK to do different things. For each question, please indicate whether *in your opinion* the answer is yes or no.

1. Do you think it is sometimes OK …
… to steal under certain circumstances?

Yes No

---

2. Do you think it is sometimes OK …
… to keep things you find in the street?

Yes No

---

3. Do you think it is sometimes OK …
… to use your fists to resolve a conflict?

Yes No

---

4. Do you think it is sometimes OK …
… to yell and argue to solve a conflict?

Yes No

---

5. Do you think it is sometimes OK …
… not to hold the door open for people?

Yes No

---

6. Do you think it is sometimes OK …
… not to tell the police when you witness a crime?

Yes No

---

7. Do you think it is sometimes OK …
… not to give directions to someone who is lost?

Yes No

---

8. Do you think it is sometimes OK …
… not to be on time for appointments?

Yes No

---

9. Do you think it is sometimes OK …
… not to return something you borrowed?

Yes No

---

10. Do you think it is sometimes OK …
… not to keep secrets that a friend told you?

Yes No

11. Do you think it is sometimes OK …
    … not to return phone calls right away?

    Yes No

12. Do you think it is sometimes OK …
    … not to spend time with friends in need?

    Yes No

Instructions:
This survey presents true/false questions about various aspects of everyday life. Please indicate, for each statement, whether you believe it to be true or false, by circling the "true" or "false". You may think that some items do not have a clear-cut answer. For those items, please try to give the answer that would be true in general, or in most cases.

Please read through the following examples to find out more about this survey.

Example:

The University of Oregon's football team is the Bruins.

We want you to do two things:

First, answer the question. In this example, you might think "*No, it's the Ducks. So the statement is* FALSE." Then you would circle 'False'.

The University of Oregon's football team is the Bruins.
This statement is [True / False].

Second, think about how sure you are of your answer. Give a number from 50% to 100%. In other words, what is the percent chance that you are right? Circle one of the numbers on the scale.

50%	60%	70%	80%	90%	100%
just guessing					*absolutely sure*

If your answer is a total guess, circle 50%. This means that there is a 50% chance that you are right, and a 50% chance that you are wrong. If you are absolutely sure, circle 100%. If you aren't sure, then circle a number in between, to show how sure you are.

In this example, you might think "*I'm absolutely sure it's false, so 100%.*" So you would circle 100%.

The University of Oregon's football team is the Bruins.
This statement is [True / False].

50%	60%	70%	80%	90%	100%
just guessing					*absolutely sure*

For each of the following statements, circle true or false to indicate your answer. Then circle a number on the scale to indicate how sure you are of your answer. The scale ranges from 50% (meaning that you were just guessing) to 100% (meaning that you were absolutely sure).

1. Many smokers use the nicotine in cigarettes to treat depression.
This statement is [True / False].

50%	60%	70%	80%	90%	100%
just guessing					*absolutely sure*

---

2. Stress makes it easier to form bad habits.
This statement is [True / False].

50%	60%	70%	80%	90%	100%
just guessing					*absolutely sure*

---

3. You can take wrinkles out of your clothes by putting them in the dryer with a damp towel.
This statement is [True / False].

50%	60%	70%	80%	90%	100%
just guessing					*absolutely sure*

---

4. There is no way to improve your memory.
This statement is [True / False].

50%	60%	70%	80%	90%	100%
just guessing					*absolutely sure*

---

5. Muscles do not burn calories when you are at rest.
This statement is [True / False].

50%	60%	70%	80%	90%	100%
just guessing					*absolutely sure*

---

6. Alcohol causes dehydration.
This statement is [True / False].

50%	60%	70%	80%	90%	100%
just guessing					*absolutely sure*

---

7. A promotion means that you will get a more satisfying job.
This statement is [True / False].

50%	60%	70%	80%	90%	100%
just guessing					*absolutely sure*

---

8. IRS forms are available on-line.
This statement is [True / False].

50%	60%	70%	80%	90%	100%
just guessing					*absolutely sure*

9. Procrastination is worse when you work in a cluttered environment.
This statement is [True / False].

50%	60%	70%	80%	90%	100%
just guessing					*absolutely sure*

---

10. Carbohydrates are fattening no matter how much you eat of them.
This statement is [True / False].

50%	60%	70%	80%	90%	100%
just guessing					*absolutely sure*

---

11. Young people face few stereotypes when looking for a job.
This statement is [True / False].

50%	60%	70%	80%	90%	100%
just guessing					*absolutely sure*

---

12. There are nonprofit organizations that help people with debt counseling.
This statement is [True / False].

50%	60%	70%	80%	90%	100%
just guessing					*absolutely sure*

---

13. Some sexually transmitted diseases can cause infertility.
This statement is [True / False].

50%	60%	70%	80%	90%	100%
just guessing					*absolutely sure*

---

14. Self-employed people pay the same amount of taxes as people who work for an employer.
This statement is [True / False].

50%	60%	70%	80%	90%	100%
just guessing					*absolutely sure*

---

15. Creating a routine is an important step in getting unpleasant work done.
This statement is [True / False].

50%	60%	70%	80%	90%	100%
just guessing					*absolutely sure*

---

16. Once you have experienced an event, your memory of it can not be changed.
This statement is [True / False].

50%	60%	70%	80%	90%	100%
just guessing					*absolutely sure*

---

17. If you get into an auto accident, let the other person take the lead in handling the details.
This statement is [True / False].

50%	60%	70%	80%	90%	100%
just guessing					*absolutely sure*

18. There is no way you can negotiate a lower rate with a credit card company.
This statement is [True / False].

50%	60%	70%	80%	90%	100%
just guessing					*absolutely sure*

---

19. Obesity increases your risk of type 2 diabetes.
This statement is [True / False].

50%	60%	70%	80%	90%	100%
just guessing					*absolutely sure*

---

20. Hard evidence is lacking that acupuncture helps you to quit smoking.
This statement is [True / False].

50%	60%	70%	80%	90%	100%
just guessing					*absolutely sure*

**Instructions:**
**Please read the practice problems on this page carefully before going on to the problems on the next page.**

Imagine Chris is going to buy a BlueRay player with the $369 he received for his birthday. He wants to find out how the BlueRay players that are available for that price compare to each other. A magazine rated BlueRay players on each of five features as follows, where higher is better:

Very Low	Low	Medium	High	Very High
1	2	3	4	5

For example, two BlueRay players and their ratings are listed in the table below:

		Features				
		Picture Quality	Sound Quality	Programming Options	Reliability of Brand	Price
BlueRay	A	2	2	5	4	$369
	B	2	3	3	3	$369

The following examples use the table above. Please read each carefully.
Example: Chris selects the BlueRay player with the highest rating in Programming Options.
Which **one** of the BlueRay players would Chris prefer? ________*A*_____

The following questions are about other people choosing between BlueRay players, like the ones above. **Please read each question carefully, because they ask for different answers.** For each question, think about how each person makes their choice, then pick the BlueRay they should choose. **Be careful, because the BlueRay players will change from question to question**.

Very Low	Low	Medium	High	Very High
1	2	3	4	5

Question 1:

		Features				
		Picture Quality	Sound Quality	Programming Options	Reliability of Brand	Price
BlueRay	A	5	4	2	1	$369
	B	5	5	3	3	$369
	C	5	2	4	4	$369
	D	1	5	5	3	$369
	E	4	5	1	1	$369

Brian selects the BlueRay player with the highest number of ratings greater than “Medium”

Which **one** of the BlueRay players would Brian prefer? ________________

Very Low	Low	Medium	High	Very High
1	2	3	4	5

Question 2:

		Features				
		Picture Quality	Sound Quality	Programming Options	Reliability of Brand	Price
BlueRay	A	2	5	5	5	$369
	B	5	4	4	5	$369
	C	5	3	2	5	$369
	D	3	5	2	2	$369
	E	4	4	4	5	$369

Sally first selects the BlueRay players with the best Sound Quality. From the selected BlueRay players, she then selects the best on Picture Quality. Then, if there is still more than one left to choose from, she selects the one best on Programming Options.

Which **one** of the BlueRay players would Sally prefer? ________________

Question 3:

		Features				
		Picture Quality	Sound Quality	Programming Options	Reliability of Brand	Price
BlueRay	A	3	1	2	5	$369
	B	5	5	3	2	$369
	C	4	3	3	3	$369
	D	5	5	5	4	$369
	E	2	5	4	4	$369

Pat doesn't want to read through the entire table. He decides to read the table row by row until he finds the very first BlueRay player that has no ratings below "Medium." He will just choose that BlueRay player.

Which **one** of the BlueRay players would Pat prefer? ________________

Question 4:

		Features				
		Picture Quality	Sound Quality	Programming Options	Reliability of Brand	Price
BlueRay	A	3	5	5	1	$369
	B	1	2	1	2	$369
	C	5	5	4	4	$369
	D	5	3	4	2	$369
	E	4	5	2	2	$369

LaToya only wants a BlueRay player that got a "Very High" rating on Reliability of Brand.

Which **one** of the BlueRay players would LaToya prefer? ________________

Very Low	Low	Medium	High	Very High
1	2	3	4	5

Question 5:

		Features				
		Picture Quality	Sound Quality	Programming Options	Reliability of Brand	Price
BlueRay	A	5	5	5	3	$369
	B	3	5	4	5	$369
	C	5	2	2	4	$369
	D	5	1	2	5	$369
	E	4	2	4	5	$369

From the BlueRay players with the best available Picture Quality, Tricia selects the BlueRay players with the lowest number of ratings below "Medium." If there is more than one BlueRay player left to choose from, she then picks the one that has the best rating on "Reliability of Brand."

Which **one** of the BlueRay players would Tricia prefer? ________________

Question 6:

		Features				
		Picture Quality	Sound Quality	Programming Options	Reliability of Brand	Price
BlueRay	A	3	1	5	2	$369
	B	1	2	1	2	$369
	C	5	4	3	1	$369
	D	4	2	3	3	$369
	E	4	4	2	4	$369

Lisa wants the BlueRay player with the highest average rating across features.

Which **one** of the BlueRay players would Lisa prefer? ________________

Question 7:

		Features				
		Picture Quality	Sound Quality	Programming Options	Reliability of Brand	Price
BlueRay	A	5	3	5	5	$369
	B	2	5	4	1	$369
	C	4	5	2	3	$369
	D	3	5	3	1	$369
	E	3	5	3	4	$369

Andy wants the BlueRay player with the highest average rating he can get while still making sure to keep the best rating on Sound Quality.

Which **one** of the BlueRay players would Andy prefer? ________________

Very Low	Low	Medium	High	Very High
1	2	3	4	5

Question 8:

		Features				
		Picture Quality	Sound Quality	Programming Options	Reliability of Brand	Price
BlueRay	A	5	4	5	3	$369
	B	5	4	1	2	$369
	C	3	3	5	5	$369
	D	5	5	1	2	$369
	E	3	5	1	3	$369

Shane wants no BlueRay players that score below "Medium" on Picture Quality, no BlueRay players that score below "Medium" on Sound Quality, and no BlueRay players that score "Very Low" on any other feature.

Which **two** of the BlueRay players would Shane prefer? _______ and _______

Question 9:

		Features				
		Picture Quality	Sound Quality	Programming Options	Reliability of Brand	Price
BlueRay	A	2	1	5	2	$369
	B	1	5	4	2	$369
	C	5	3	1	1	$369
	D	5	4	5	4	$369
	E	3	3	3	3	$369

Tyrone wants a BlueRay player that either has a "Very High" rating for Programming Options, or one that scores at least "Medium" on every feature.

Which **three** of the BlueRay players would Tyrone prefer? _______, _______, and _______

Question 10:

		Features				
		Picture Quality	Sound Quality	Programming Options	Reliability of Brand	Price
BlueRay	A	2	1	5	4	$369
	B	4	5	1	3	$369
	C	1	3	5	5	$369
	D	4	2	5	4	$369
	E	5	5	1	3	$369

Julie wants the best Reliability of Brand, but is willing to give up one point on Reliability of Brand for each increase of at least two points in the rating of Picture Quality. She isn't concerned about the other features.

Which **three** of the BlueRay players would Julie prefer? _______, _______, and _______

Instructions:
Each of these questions asks for your best guess at the chance that something will happen in the future. They use the "probability" scale that you see below. To answer each question, please put a mark on the scale at one specific tick mark, as follows:

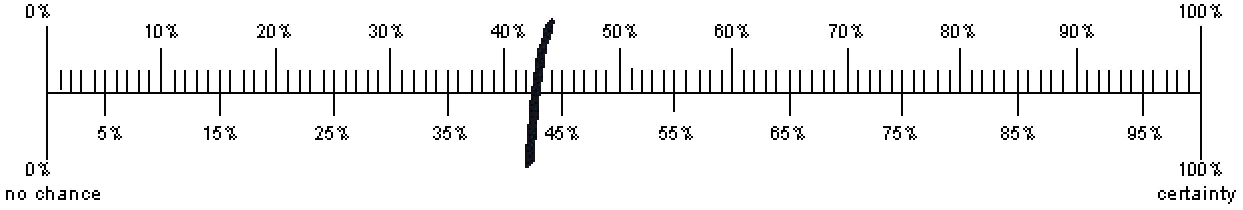

If you think that something has no chance of happening, mark it as having a 0% chance. If you think that something is certain to happen, mark it as having a 100% chance.

Just to make sure that you are comfortable with the scale, please answer the following practice questions.

What is the probability that you will eat pizza during the next year?

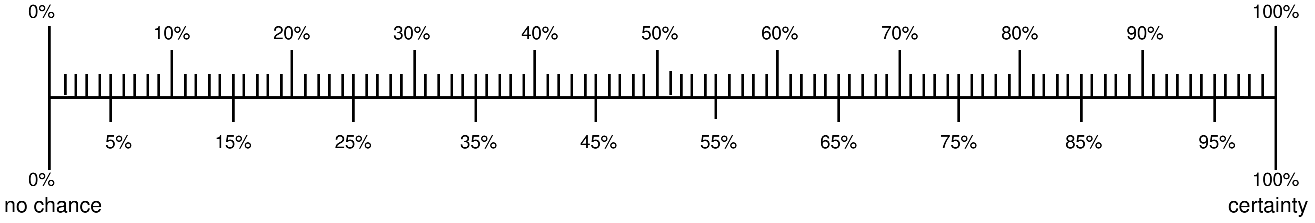

That is the end of the practice. If you have any questions, please ask them now.

A. The following questions ask about events that may happen some time during *the next year*.

1. What is the probability that you will get into a car accident while driving during the next year?

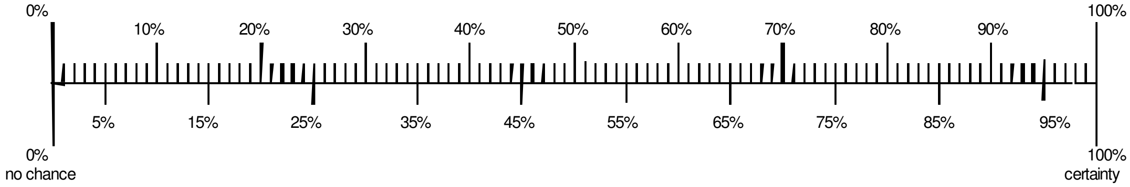

2. What is the probability that you will have a cavity filled during the next year?

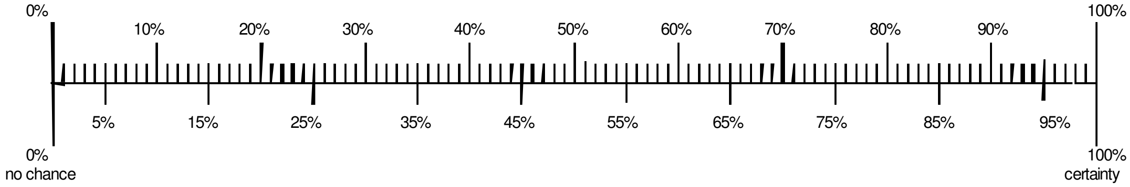

3. What is the probability that you will die (from any cause -- crime, illness, accident, and so on) during the next year?

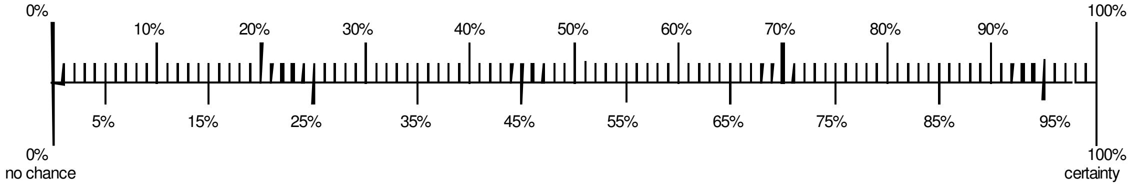

4. What is the probability that someone will steal something from you during the next year?

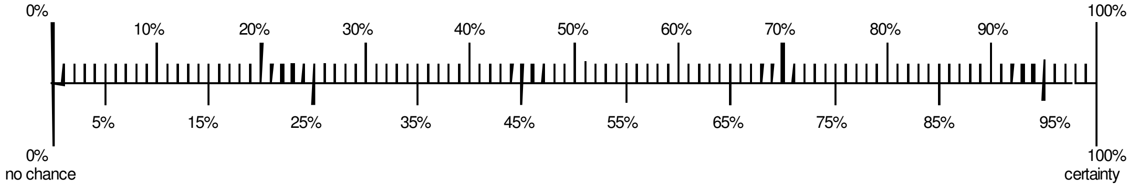

5. What is the probability that you will move your permanent address to another state some time during the next year?

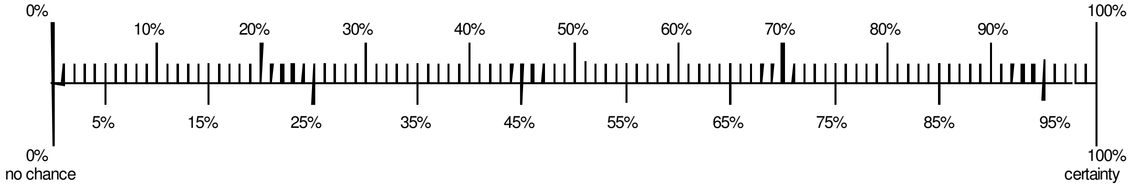

6. What is the probability that you will die in a terrorist attack during the next year?

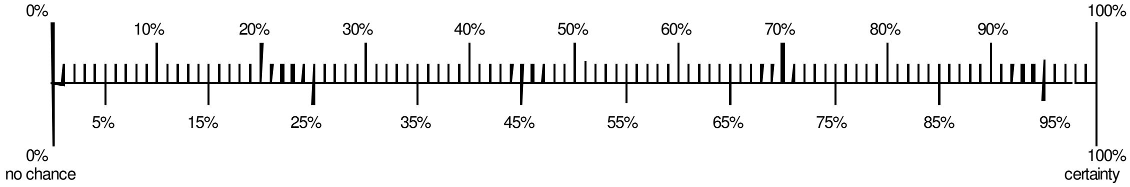

7. What is the probability that someone will break into your home and steal something from you during the next year?

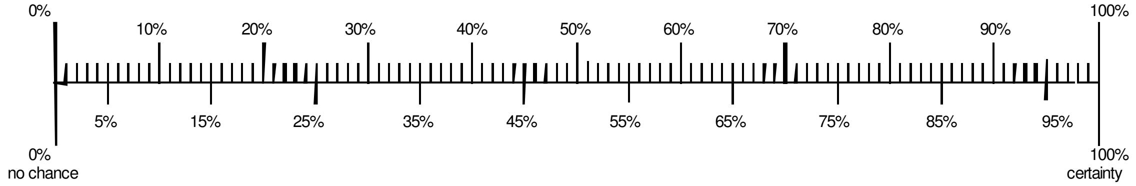

8. What is the probability that you will keep your permanent address in the same state during the next year?

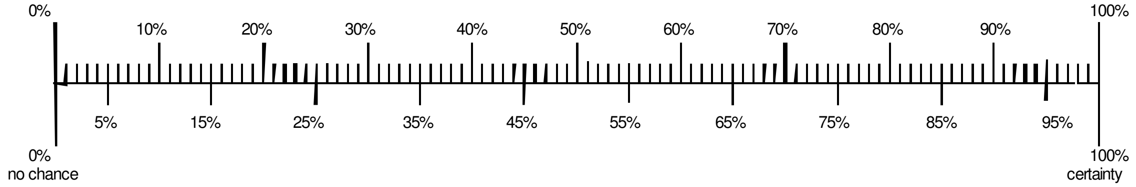

9. What is the probability that you will visit a dentist, for any reason, during the next year?

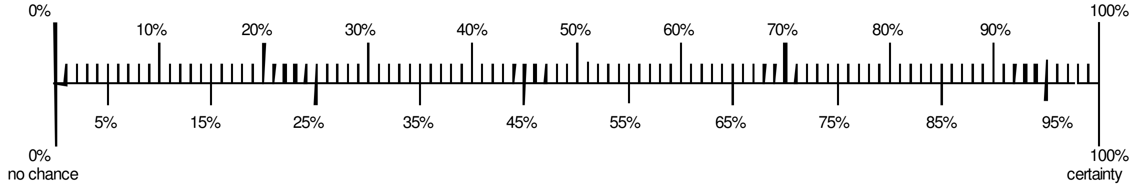

10. What is the probability that your driving will be accident-free during the next year?

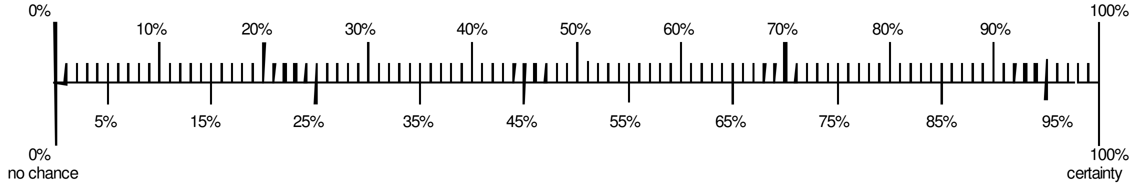

B. The following questions ask about events that may happen some time during *the next 5 years*.

1. What is the probability that you will get into a car accident while driving during the next 5 years?

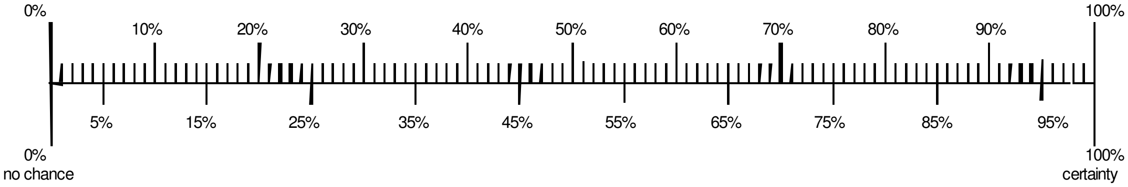

2. What is the probability that you will have a cavity filled during the next 5 years?

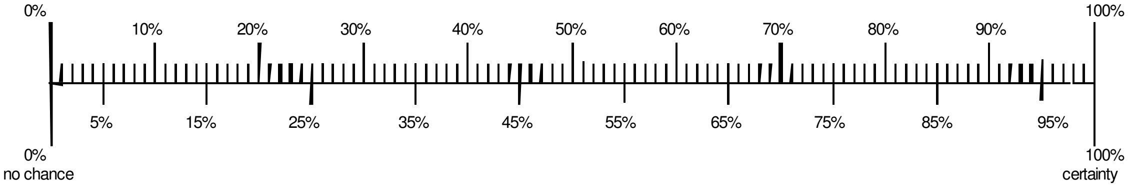

3. What is the probability that you will die (from any cause -- crime, illness, accident, and so on) during the next 5 years?

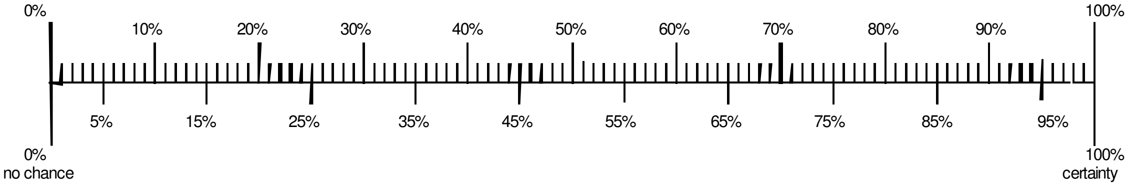

4. What is the probability that someone will steal something from you during the next 5 years?

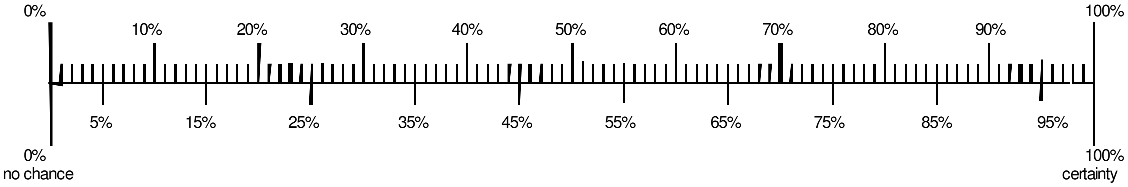

5. What is the probability that you will move your permanent address to another state some time during the next 5 years?

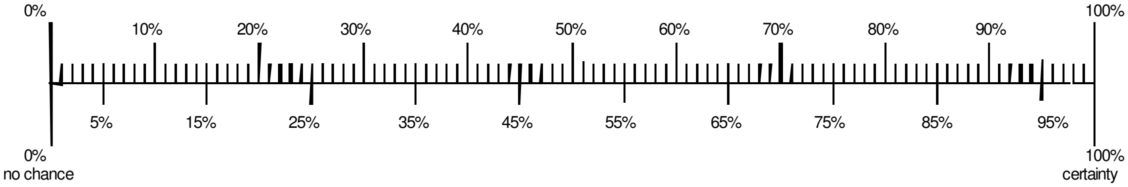

6. What is the probability that you will die in a terrorist attack during the next 5 years?

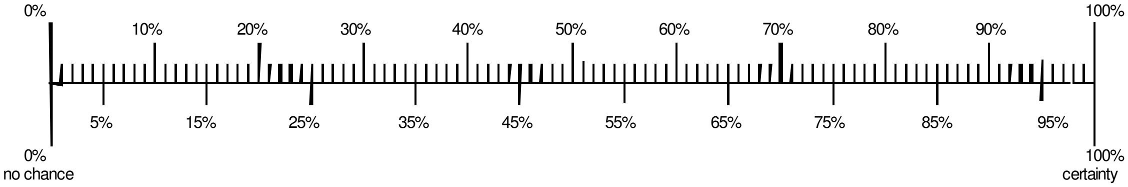

7. What is the probability that someone will break into your home and steal something from you during the next 5 years?

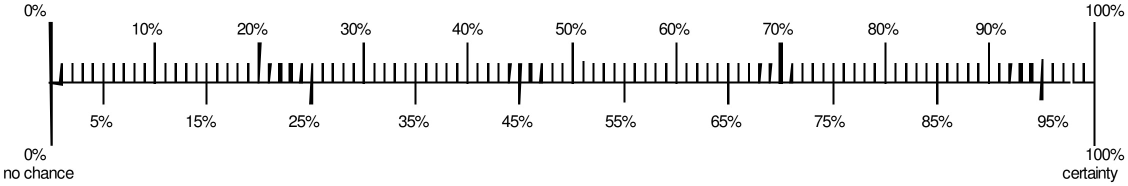

8. What is the probability that you will keep your permanent address in the same state during the next 5 years?

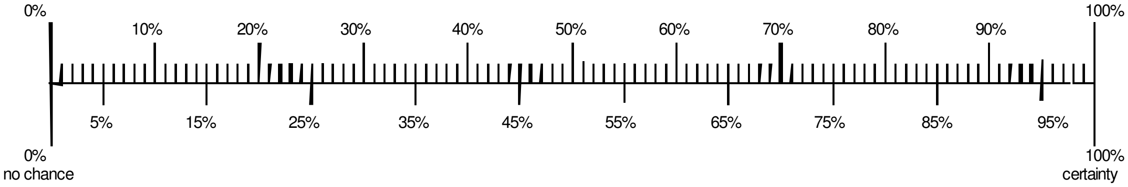

9. What is the probability that you will visit a dentist, for any reason, during the next 5 years?

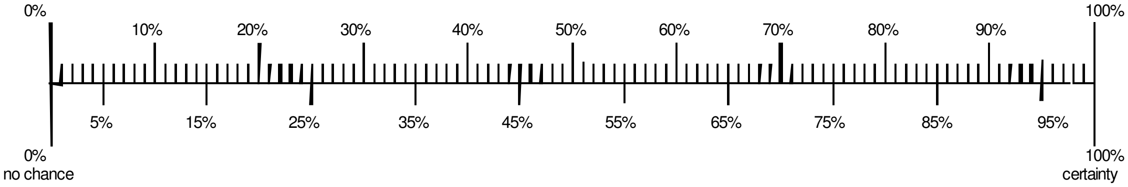

10. What is the probability that your driving will be accident-free during the next 5 years?

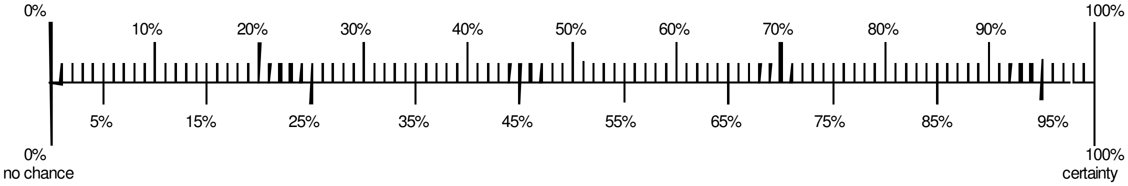

<u>Instructions:</u>
Each of the following problems presents a choice between two options. Each problem is presented with a scale ranging from 1 (representing one option) through 6 (representing the other option). For each item, please circle the number on the scale that best reflects your relative preference between the two options.

Problem 1
You are buying a gold ring on layaway for someone special. It costs $200 and you have already paid $100 on it, so you owe another $100. One day, you see in the paper that a new jewelry store is selling the same ring for only $90 as a special sale, and you can pay for it using layaway. The new store is across the street from the old one. If you decide to get the ring from the new store, you will not be able to get your money back from the old store, but you would save $10 overall.

Would you be more likely to continue paying at the old store or buy from the new store?

1	2	3	4	5	6
Most likely to continue paying at the old store					*Most likely to buy from the new store*

Problem 2
You enjoy playing tennis, but you really love bowling. You just became a member of a tennis club, and of a bowling club, both at the same time. The membership to your tennis club costs $200 per year and the membership to your bowling club $50 per year. During the first week of both memberships, you develop an elbow injury. It is painful to play either tennis or bowling. Your doctor tells you that the pain will continue for about a year.

Would you be more likely to play tennis or bowling in the next six months?

1	2	3	4	5	6
Most likely to play tennis					*Most likely to play bowling*

Problem 3
You have been looking forward to this year’s Halloween party. You have the right cape, the right wig, and the right hat. All week, you have been trying to perfect the outfit by cutting out a large number of tiny stars to glue to the cape and the hat, and you still need to glue them on. On the day of Halloween, you decide that the outfit looks better without all these stars you have worked so hard on.

Would you be more likely to wear the stars or go without?

1	2	3	4	5	6
Most likely to wear stars					*Most likely to not wear stars*

Problem 4
After a large meal at a restaurant, you order a big dessert with chocolate and ice cream. After a few bites you find you are full and you would rather not eat any more of it.

Would you be more likely to eat more or to stop eating it?

1	2	3	4	5	6
Most likely to eat more					*Most likely to stop eating*

Problem 5
You are in a hotel room for one night and you have paid $6.95 to watch a movie on pay TV. Then you discover that there is a movie you would much rather like to see on one of the free cable TV channels. You only have time to watch one of the two movies.

Would you be more likely to watch the movie on pay TV or on the free cable channel?

1	2	3	4	5	6
Most likely to watch pay TV					*Most likely to watch free cable*

Problem 6
You have been asked to give a toast at your friend's wedding. You have worked for hours on this one story about you and your friend taking drivers' education, but you still have some work to do on it. Then you realize that you could finish writing the speech faster if you start over and tell the funnier story about the dance lessons you took together.

Would you be more likely to finish the toast about driving or rewrite it to be about dancing?

1	2	3	4	5	6
Most likely to write about driving					*Most likely to write about dancing*

Problem 7
You decide to learn to play a musical instrument. After you buy an expensive cello, you find you are no longer interested. Your neighbor is moving and you are excited that she is leaving you her old guitar, for free. You'd like to learn how to play it.

Would you be more likely to practice the cello or the guitar?

1	2	3	4	5	6
Most likely to play cello					*Most likely to play guitar*

Problem 8
You and your friend are at a movie theater together. Both you and your friend are getting bored with the storyline. You'd hate to waste the money spent on the ticket, but you both feel that you would have a better time at the coffee shop next door. You could sneak out without other people noticing.

Would you be more likely to stay or to leave?

1	2	3	4	5	6
Most likely to stay					Most likely to leave

Problem 9
You and your friend have driven halfway to a resort. Both you and your friend feel sick. You both feel that you both would have a much better weekend at home. Your friend says it is "too bad" you already drove halfway, because you both would much rather spend the time at home. You agree.

Would you be more likely to drive on or turn back?

1	2	3	4	5	6
Most likely to drive on					*Most likely to turn back*

Problem 10
You are painting your bedroom with a sponge pattern in your favorite color. It takes a long time to do. After you finish two of the four walls, you realize you would have preferred the solid color instead of the sponge pattern. You have enough paint left over to redo the entire room in the solid color. It would take you the same amount of time as finishing the sponge pattern on the two walls you have left.

Would you be more likely to finish the sponge pattern or to redo the room in the solid color?

1	2	3	4	5	6
Most likely to finish sponge pattern					*Most likely to redo with a solid color*

Instructions:
Each of the following problems presents a choice between two options. Each problem is presented with a scale ranging from 1 (representing one option) through 6 (representing the other option). For each item, please circle the number on the scale that best reflects your relative preference between the two options.

Problem 1

Imagine a hospital is treating 32 injured soldiers, who are all expected to lose one leg. There are two doctors that can help the soldiers, but only one can be hired:

If Doctor A is hired, 12 soldiers will lose one leg.

If Doctor B is hired, there is a 63% chance that nobody loses a leg and a 37% chance that all lose a leg.

Which doctor do you recommend?

1	2	3	4	5	6
Definitely would choose A					*Definitely would choose B*

Problem 2

Imagine that the U.S. is preparing for the outbreak of an unusual disease, which is expected to kill 600 people. Two alternative programs to combat the disease have been proposed. Assume that the exact scientific estimates of the consequences of the programs are as follows:

If Program A is adopted, 400 people will die.

If Program B is adopted, there is a 33% chance that nobody will die, and a 67% chance that 600 people will die.

Which program do you recommend to use?

1	2	3	4	5	6
Definitely would choose A					*Definitely would choose B*

Problem 3

Imagine that your client has $6,000 invested in the stock market. A downturn in the economy is occurring. You have two investment strategies that you can recommend under the existing circumstances to preserve your client's capital.

If strategy A is followed, $4,000 of your client's investment will be lost.

If strategy B is followed, there is a 33% chance that the nothing will be lost, and a 67% chance that $6,000 will be lost.

Which of these two strategies would you favor?

1	2	3	4	5	6
Definitely would choose A					*Definitely would choose B*

Problem 4

Because of changes in tax laws, you may get back as much as $1200 in income tax. Your accountant has been exploring alternative ways to take advantage of this situation. He has developed two plans:

If Plan A is adopted, you will lose $800 of the possible $1200.

If Plan B is adopted, you have a 33% chance of losing none of the money, and a 67% chance of losing all $1200.

Which plan would you use?

1	2	3	4	5	6
Definitely would choose A					*Definitely would choose B*

Problem 5

Imagine that recent evidence has shown that a pesticide is threatening the lives of 1,200 endangered animals. Two response options have been suggested:

If Option A is used, 600 animals will be lost for sure.

If Option B is used, there is a 75% chance that 400 animals will be lost, and a 25% chance that 1,200 animals will be lost.

Which option do you recommend to use?

1	2	3	4	5	6
Definitely would choose A					*Definitely would choose B*

Problem 6

Imagine that your doctor tells you that you have a cancer that must be treated. Your choices are as follows:

Surgery: Of 100 people having surgery, 10 die because of the operation, and 66 die by the end of five years.

Radiation therapy: Of 100 people having radiation therapy, none die during the treatment, and 78 die by the end of five years.

Which treatment would you choose?

1	2	3	4	5	6
Definitely would choose surgery					*Definitely would choose radiation*

Problem 7

Imagine that in one particular state it is projected that 1000 students will drop out of school during the next year. Two programs have been proposed to address this problem, but only one can be implemented. Based on other states' experiences with the programs, estimates of the outcomes that can be expected from each program can be made. Assume for purposes of this decision that these estimates of the outcomes are accurate and are as follows:

If Program A is adopted, 600 of the 1000 students will drop out of school.

If Program B is adopted, there is a 40% chance that none of the 1000 students will drop out of school and 60% chance that all 1000 students will drop out of school.

Which program would you favor for implementation?

1	2	3	4	5	6
Definitely would choose A					*Definitely would choose B*

Instructions:

Each of the following problems ask you to rate your judgment of a product or a situation. Each problem is presented with a scale ranging from 1 (representing the worst rating) through 6 (representing the best rating). For each problem, please circle the number on the scale that best reflects your judgment.

Problem 1

As manager, one of your project teams has come to you requesting an additional $100,000 in funds for a project you instituted several months ago. The project is already behind schedule and over budget, but the team still believes it can be successfully completed. You currently have $500,000 remaining in your budget unallocated, but which must carry you for the rest of the fiscal year. Lowering the balance by an additional $100,000 might jeopardize flexibility to respond to other opportunities.

Evaluating the situation, you believe there is a fair chance the project will not succeed, in which case the additional funding would be lost; if successful, however, the money would be well spent. You also noticed that of the projects undertaken by this team, 20 of the last 50 have been unsuccessful.

What is the likelihood you would fund the request?

1	2	3	4	5	6
Very unlikely					*Very likely*

Problem 2

Imagine that a woman parked illegally. After talking to her, you believe that there is an 80% chance that she knew she parked illegally.

With this in mind, how much of a fine do you believe this woman deserves?

1	2	3	4	5	6
Minimum fine					*Maximum fine*

Problem 3

In a recent confidential survey completed by graduating seniors, 65% of those completing the survey stated that they had cheated during their college career.

Considering the results of the survey, how would you rate the incidence of cheating at your university?

1	2	3	4	5	6
Very low					*Very high*

Problem 4

Imagine that a new technique has been developed to treat a particular kind of cancer. This technique has a 50% chance of failure, and is available at the local hospital.

A member of your immediate family is a patient at the local hospital with this kind of cancer. How likely are you to encourage him or her to undergo treatment using this technique?

1	2	3	4	5	6
Definitely no					*Definitely yes*

Problem 5

Imagine the following situation. You are entertaining a special friend by inviting them for dinner. You are making your favorite lasagna dish with ground beef. Your roommate goes to the grocery store and purchases a package of ground beef for you. The label says 20% fat ground beef.

What's your evaluation of the quality of this ground beef?

1	2	3	4	5	6
Very low					*Very high*

Problem 6

Suppose a student got 10% incorrect in the mid-term exam and 30% incorrect in the final-term exam, what would be your evaluations of this student's performance?

1	2	3	4	5	6
Very poor					*Very good*

<u>Instructions:</u>
The following problems ask *out of 100 people your age*, how many would say that it is sometimes OK to do different things. For each question, please circle a number between 0 (meaning *no one* thinks that it is sometimes OK) and 100 (meaning *everyone* thinks that it is sometimes OK).

1. Out of 100 people your age, how many would say it is sometimes OK …
… to steal under certain circumstances?

0 10 20 30 40 50 60 70 80 90 100
No one *Everyone*

---

2. Out of 100 people your age, how many would say it is sometimes OK …
… to smoke cigarettes?

0 10 20 30 40 50 60 70 80 90 100
No one *Everyone*

---

3. Out of 100 people your age, how many would say it is sometimes OK …
… to commit a crime which could put you in jail?

0 10 20 30 40 50 60 70 80 90 100
No one *Everyone*

---

4. Out of 100 people your age, how many would say it is sometimes OK …
… to keep things you find in the street?

0 10 20 30 40 50 60 70 80 90 100
No one *Everyone*

---

5. Out of 100 people your age, how many would say it is sometimes OK …
… to experiment with marijuana?

0 10 20 30 40 50 60 70 80 90 100
No one *Everyone*

---

6. Out of 100 people your age, how many would say it is sometimes OK …
… to use your fists to resolve a conflict?

0 10 20 30 40 50 60 70 80 90 100
No one *Everyone*

---

7. Out of 100 people your age, how many would say it is sometimes OK …
… to drink and drive?

0 10 20 30 40 50 60 70 80 90 100
No one *Everyone*

---

8. Out of 100 people your age, how many would say it is sometimes OK …
… to yell and argue to solve a conflict?

0 10 20 30 40 50 60 70 80 90 100
No one *Everyone*

9. Out of 100 people your age, how many would say it is sometimes OK …
… not to hold the door open for people?

0	10	20	30	40	50	60	70	80	90	100
No one										*Everyone*

10. Out of 100 people your age, how many would say it is sometimes OK …
… not to tell the police when you witness a crime?

0	10	20	30	40	50	60	70	80	90	100
No one										*Everyone*

11. Out of 100 people your age, how many would say it is sometimes OK …
… not to give directions to someone who is lost?

0	10	20	30	40	50	60	70	80	90	100
No one										*Everyone*

12. Out of 100 people your age, how many would say it is sometimes OK …
… not to be on time for appointments?

0	10	20	30	40	50	60	70	80	90	100
No one										*Everyone*

13. Out of 100 people your age, how many would say it is sometimes OK …
… not to return something you borrowed?

0	10	20	30	40	50	60	70	80	90	100
No one										*Everyone*

14. Out of 100 people your age, how many would say it is sometimes OK …
… not to keep secrets that a friend told you?

0	10	20	30	40	50	60	70	80	90	100
No one										*Everyone*

15. Out of 100 people your age, how many would say it is sometimes OK …
… not to return phone calls right away?

0	10	20	30	40	50	60	70	80	90	100
No one										*Everyone*

16. Out of 100 people your age, how many would say it is sometimes OK …
… not to spend time with friends in need?

0	10	20	30	40	50	60	70	80	90	100
No one										*Everyone*
